# High-throughput immuno-profiling of mamba (*Dendroaspis*) venom toxin epitopes using high-density peptide microarrays

**DOI:** 10.1038/srep36629

**Published:** 2016-11-08

**Authors:** Mikael Engmark, Mikael R. Andersen, Andreas H. Laustsen, Jigar Patel, Eric Sullivan, Federico de Masi, Christian S. Hansen, Jens V. Kringelum, Bruno Lomonte, José María Gutiérrez, Ole Lund

**Affiliations:** 1 Technical University of Denmark, Department of Bio and Health Informatics, Kgs. Lyngby, 2800, Denmark; 2 Technical University of Denmark, Department of Biotechnology and Biomedicine, Kgs. Lyngby, 2800, Denmark; 3 University of Copenhagen, Department of Drug Design and Pharmacology, Faculty of Health and Medical Sciences, Copenhagen East, 2100, Denmark; 4 Roche NimbleGen, Madison, Wisconsin 53719, USA; 5 Instituto Clodomiro Picado, Facultad de Microbiología, Universidad de Costa Rica, San José 11501, Costa Rica

## Abstract

Snakebite envenoming is a serious condition requiring medical attention and administration of antivenom. Current antivenoms are antibody preparations obtained from the plasma of animals immunised with whole venom(s) and contain antibodies against snake venom toxins, but also against other antigens. In order to better understand the molecular interactions between antivenom antibodies and epitopes on snake venom toxins, a high-throughput immuno-profiling study on all manually curated toxins from *Dendroaspis* species and selected African *Naja* species was performed based on custom-made high-density peptide microarrays displaying linear toxin fragments. By detection of binding for three different antivenoms and performing an alanine scan, linear elements of epitopes and the positions important for binding were identified. A strong tendency of antivenom antibodies recognizing and binding to epitopes at the functional sites of toxins was observed. With these results, high-density peptide microarray technology is for the first time introduced in the field of toxinology and molecular details of the evolution of antibody-toxin interactions based on molecular recognition of distinctive toxic motifs are elucidated.

Snakebite envenoming is a much neglected tropical health condition, affecting more than 2.5 million victims per year in mainly rural settings across the poorest regions of the World^1^. Parenteral administration of antivenom has since its development in the 1890’s constituted the only effective treatment for snakebite envenomings^2^. One of the reasons for the paucity of breakthrough innovations in the field of antivenom research and development may be that snake venoms are among the most complex drug targets known, owing to their great biochemical diversity^1^,^3^. Moreover, antivenoms are complex medicines, consisting of polyclonal mixtures of immunoglobulins or fragments thereof, which are produced following traditional protocols involving repeated animal immunisation with venoms. During the immunisation process, a diverse pool of antibodies is raised, and these antibodies bind to and neutralise toxins and non-toxic components present in the venoms^4^,^5^. Despite the clinical importance of many snake venoms, little is known about the specific interactions between antivenom antibodies and snake venom toxins^6^,^7^.

At the venom level, an increasing number of studies, based on proteomics and referred to as “antivenomics”, are being performed^8^,^9^. These investigations provide valuable information on which venom components are recognised by antibodies from a given antivenom. Moreover, the antivenomics protocol allows for investigation of antivenom para-specificity (i.e. cross-reactivity to snake venom toxins not included in the immunisation mixture) and can thereby be used for the rational design, development, and clinical use of antivenoms^9^,^10^. Although the antivenomics methodology has proven to be a useful tool for descriptive purposes, it does not provide deeper insights into the interactions between toxins and antibodies at the molecular level and thereby explain the origin of any observed venom para-specificity.

Studies of snake toxin epitopes have traditionally been performed as time-consuming cross-reactivity experiments on only about a dozen of model toxins, deriving mainly from the group of elapid α-neurotoxins^11^. Recently, studies involving immunoassay quantification of antivenom binding to immobilised synthetic peptides (SPOT synthesis), corresponding to individual segments of the amino acid sequence of a given toxin, have added valuable molecular insight by elucidating which sequences contain linear elements of epitopes recognised by given antivenoms^6^,^7^,^12^,^13^,^14^. These kind of meticulous epitope mapping experiments have also been performed on toxins from *Tityus* scorpions^15^,^16^,^17^ and *Loxosceles* spiders^18^. The possibility of combining antivenomic assessment of antivenoms with a high-throughput epitope mapping approach of snake venom toxins may provide deeper insight into toxin-antivenom interactions. Such insight may further be used to understand the basis of antivenom specificity and para-specificity, which may guide the development of broad-acting antivenoms with higher efficacy and polyvalence.

In this study, we focused on venom toxins from the four species-membered *Dendroaspis* (mamba) genus, a group of snakes in sub-Saharan Africa of the highest medical importance. Mamba envenomings are known for their rapid onset of neurotoxicity^19^,^20^, which may manifest itself already after 15 min via clinical signs such as flaccid paralysis, dyspnea due to respiratory muscle paralysis, and involuntary skeletal muscle contractions or fasciculations^19^. These effects are explained by the venom compositions, which are dominated by potent small neurotoxins belonging to the three-finger toxin family and Kunitz-type serine protease inhibitor family^21^,^22^,^23^. α-neurotoxins bind to nicotinic cholinergic receptors at the motor end-plate of muscle fibers, thereby blocking neuromuscular transmission^24^. Dendrotoxins, which belong to the Kunitz-type serine protease inhibitor protein family, interact with voltage-dependent potassium channels, leading to excitatory effects due to facilitation of the release of acetylcholine and potentiation of its effect at the presynaptic nerve terminal^25^. In addition, fasciculins, which are inhibitors of acetylcholinesterase (AChE), have been isolated from *D. angusticeps* venom, and these quite unique AChE inhibitors may induce increased synaptic concentrations of acetylcholine causing fasciculation^26^. Although many toxins have been isolated and sequenced from *Dendroaspis* venoms, only the venoms of *D. polylepis* and *D. angusticeps* have so far undergone a thorough toxicovenomics analysis^21^,^22^. In spite of the significant differences in venom compositions, many similar toxins exist in *Dendroaspis* venoms, although in different relative proportions, which is to be expected given the phylogenetic proximity of the four species^27^.

The present study aims at exploring the binding patterns of three polyvalent antivenoms distributed in sub-Saharan Africa, which include mamba venoms as part of their immunizing mixtures, towards the 61 mamba venom proteins available in the UniProtKB database^28^. Using state-of-the art SPOT strategy this would comprise the synthesis of 1,026 unique 12-mer peptides. To tackle this challenge, we introduce high-density peptide microarray technology^29^ for the first time in the field of antivenom research. Overlapping 12-mer peptides, covering the entire primary sequence of each neurotoxin, were prepared by light-directed solid-phase synthesis. 21 neurotoxins from three African *Naja* (cobra) species were also included on the high-density peptide microarray. These related neurotoxins were included to address potential convoluted epitope preferences of antivenom resulting from inclusion of venom(s) from African cobra snakes in the immunisation mixture for the antivenoms. This setup allowed an investigation of antivenom cross-recognition of linear epitopes among the toxins. Moreover, mutated versions of 12-mers corresponding to single replacement of each amino acid with alanine (i.e. alanine scanning) were additionally included, aiming to identify key residues in epitopes responsible for antibody binding. This dramatically increased the number of unique synthesised peptides with 20,056.[Table t1]

## Results and Discussion

Aiming at comprehensively mapping all epitopic linear elements in the 82 mamba and cobra venom toxins in Tables 2 and 3 on the basis of recognition by three therapeutic antivenoms, an approach built upon custom-made high-throughput peptide microarrays was employed. All antivenoms consisted of F(ab′)_2_ fragments obtained by pepsin digestion of IgG antibodies from equine serum. The reported immunisation mixtures for the hyper-immunised horses are summarised in Table 1. Each antivenom was employed in two different dilutions of the original samples, resulting in a total of six peptide microarray experiments.

**Figure 1:**
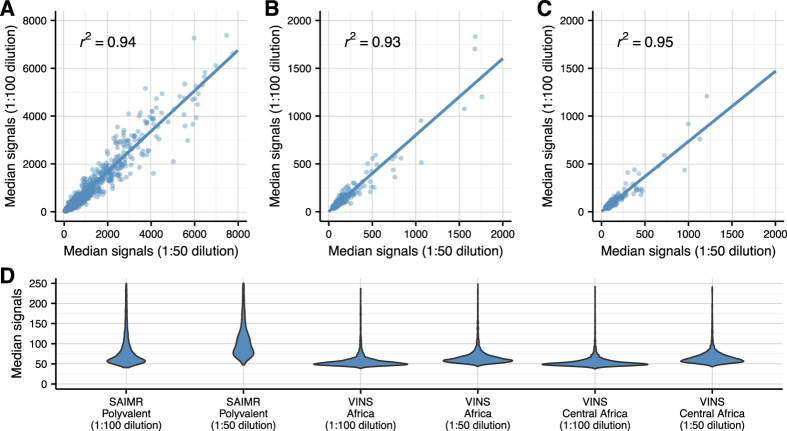
Correlation between experiments with the same antivenom used in different dilutions show a high degree of reproducibility and agreement between the microarray experiments. The correlation of median signal intensities of five replicates of each peptide is plotted for each pair of experiments conducted with the same antivenom. **(A)** SAIMR Polyvalent Snake Antivenom, **(B)** VINS African, and **(C)** VINS Central Africa. **(D)** Violin plots illustrating the distribution of peptide-specific median signals below 250 AU for each experiment.

Peptide microarray experiments conducted with the same antivenom in different dilutions were in good agreement with each other, as shown in Fig. 1A–C. In each experiment, the vast majority of peptides were found to provide low signal intensities. This is displayed as violin plots in Fig. 1D and it can be seen that the majority of peptides have unspecific binding or auto-fluorescence following a normal distribution. Although a smooth transition is observed between the background signals and signals resulting from putative antibody-binding, it is easy to identify peptides with a high signal-to-noise ratio even by visual inspection. Comparing the six violin plots in Fig. 1D, it is also evident that the distribution of background signals is not directly comparable among antivenoms from different producers (SAVP and VINS Bioproducts). For the SAIMR Polyvalent Snake Antivenom from SAVP, much higher signals were observed than for the VINS products (see also Fig. 1A–C). This bias is likely due to differences in antivenom formulations and production methods, as well as the concentration of antibody fragments in the formulation (see Table 1).

Key findings within the most important toxin families in mamba and cobra venoms are presented in the following subsections, where focus is directed on toxins of high medical importance as judged by their Toxicity Scores determined in previous studies^21^,^22^.

### Mapping of antibody binding reveals antigenic hotspots in toxin sequences

**Figure 2:**
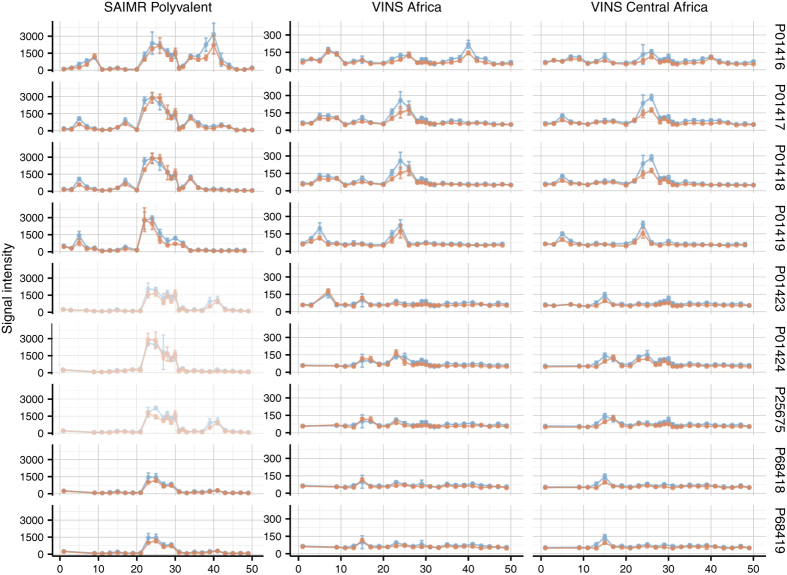
Binding profiles of nine type 1 α-neurotoxins (a subfamily of the short chain three-finger toxin subfamily). The median signal intensity of five replicates of each peptide is plotted as dots based on the position of the N-terminal residue of the 12-mer peptide in the protein sequences (pro-peptides removed). The dots are connected with straight lines to visualise the relation between overlapping peptides. The error bars represent the standard deviation of the five replicates. Positions containing gaps in the alignments were ignored. Blue dots refer to the 1:50 dilution experiment, while red dots refer to the 1:100 dilution experiments. Notice that many 12-mer peptides are present in several individual toxin entries and that each point contains information on the following 11 amino acids of the toxin. Binding profiles of all 82 toxins in this study are found in Supplementary Figs S1–S7.

Linear elements in epitopes are usually between 7 and 9 amino acid residues in size^30^,^31^ implying that a high signal is likely to be observed across overlapping 12-mer peptides containing the epitope motif. Applying this principle, signal intensities for each of the peptides were mapped back to the respective toxin sequences from which the peptides were derived. By plotting signal intensity as a function of peptide position in the toxin sequence, binding profiles of each toxin-antivenom pair were obtained. The binding profiles of the type 1 α-neurotoxins included in the study can be found in Fig. 2 for illustration of how peaks in signal intensity, corresponding to areas containing linear epitopic elements, are clearly distinguished from the background noise. For each antivenom, two binding profiles corresponding to the two separate experiments with different dilutions were obtained. As shown in Fig. 2, the two sets of results (coloured blue and red) were found to give similar binding profiles reflecting a high degree of reproducibility of results even when the dilutions were altered (Fig. 1A–C). Moreover, the binding profiles for the type 1 α-neurotoxins display a general tendency for all three antivenoms to peak around peptide number 25 (covering the 12 residues from 25 to 36). A more in-depth discussion on the antibody recognition of the type 1 α-neurotoxins is reserved for a later section. The complete collection of binding profiles for the 82 investigated mamba and cobra toxins can be found in Supplementary Figs S1–7.

### A high number of binding peptides were identified with SAIMR polyvalent antivenom

The numeric size of the signal for a peptide being recognised by the polyvalent antivenoms is a complex function of the concentration of one or more specific antibodies, the affinity of each of the antibodies, and the structural arrangement and conformation of the peptide on the microarray^30^. Also, variation in peptide solvation is likely to play a role. Regardless of the molecular origin of signals for peptides recognised by antibodies, it is crucial to separate them from the noise resulting from unspecific binding and auto-fluorescence. For each experiment, putative epitope-containing peptides were identified based on the median signal intensity of each peptide and the assumption that the majority of peptides were not recognised by the antibodies. The assumption is based on the distribution of all signals (Fig. 1), supported by the presence of clear baselines in the binding profiles (Fig. 2 and Supplementary Figs S1–7), and it is in agreement with the findings in a previous study with antivenom and members of the three-finger toxin protein family^12^. As a conservative estimate, the lower 70-percentile of median signals was used to determine the mean and standard deviations of the background populations, and a peptide was classified as target for antivenom antibodies if the median signal intensity was more than 10 standard deviations above the mean (see method section for details).

**Figure 3:**
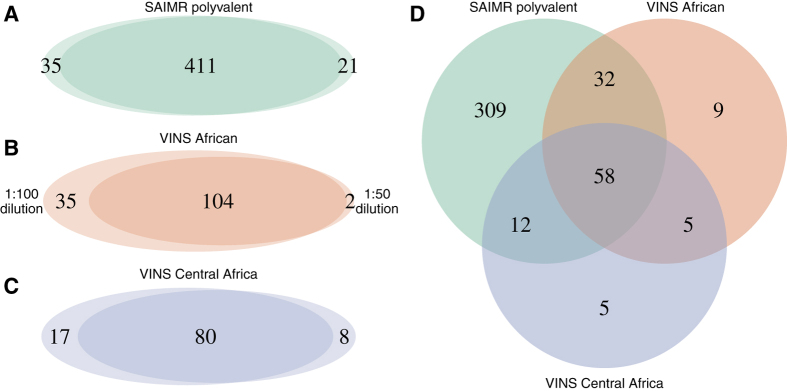
(**A–C**) Venn diagrams of peptides classified to bind antivenom antibodies for each pair of experiments conducted with the same antivenom in two different dilutions; **(A)** SAIMR Polyvalent Snake Antivenom, **(B)** VINS African, and **(C)** VINS Central Africa. **(D)** Venn diagram of peptides classified as binders for each antivenom. Only peptides identified in both experiments with each antivenom, corresponding to the overlap in Venn diagram in part A–C, are included.

The overlap between the groups of peptides classified as antibody binders in the individual experiments was determined and the outcome is shown as Venn diagrams in Fig. 3. For microarrays with the same antivenom in different dilutions, the majority of the identified binders is shared within the data sets. Experiments employing the SAIMR polyvalent antivenom at two different dilutions displayed the highest degree of agreement between two experiments with only 12% non-shared peptides, while the number of non-shared peptides were 26% and 20% for for VINS African antivenom and VINS Central Africa antivenom, respectively (Fig. 3A–C). The two VINS antivenoms were found to be relatively similar in their recognition of the peptides, although VINS African antivenom recognises a higher number of peptides than VINS Central Africa antivenom (Fig. 3D). Based on the reported immunisation mixtures in Table 1, the broader recognition of peptides from elapid toxins by VINS African compared to VINS Central Africa is in agreement with the broader range of venoms that the antivenom is raised against (Table 1).

Considering only the peptides displaying antibody binding at both concentrations for each antivenom, a considerable fraction consisting of 58 unique peptides was found to be recognised by all three antivenoms. Also, only 19 unique peptides were found to bind one or both of the VINS antivenoms and not the SAIMR antivenom (Fig. 3D). In fact, the SAIMR antivenom was found to bind four times as many peptides as the two antivenoms from VINS Bioproducts. Also, binding was observed between the SAIMR antivenom and one or more peptides for 80 out of the 82 toxins included in the study. Only the two short chain three-finger toxins, Weak toxin CM-2 (P01415) from *N. haje* and a fragment of Muscarinic m1-toxin3 (P60235) from *D. angusticeps*, were not recognised.

Due to the low number of peptides recognised exclusively by the VINS antivenoms and the more distinct signals in the microarrays loaded with SAIMR antivenom, the epitope characterisation in the following sections is based on the results from the SAIMR antivenom only.

### Alanine substitution analysis reveals key residues for antivenom toxin recognition

For all 12-mers classified as antibody-binding peptides, the decrease or increase in signal intensity of replacing each residue for alanine was determined. This had the goal of identifying residues of importance for the antibody-toxin interaction, as the substitution for alanine is thought to be a good model for removing single functionalities, one at a time, from a given peptide. An average alanine substitution effect was calculated for each amino acid, taking the substitution effects at multiple positions in the 12-mer setup into account. This means that, when an amino acid is present on multiple overlapping antibody-binding 12-mers, the effect of the position of the residue in each single peptide (e.g. in the C-terminal or N-terminal part) is reduced.

As the average alanine substitution effect of a given residue is a relative number, it does not reflect the size of the original signal intensities. Therefore, the binding profiles (Fig. 2 and Supplementary Figs S1–7) were configured to be a function of the amino acid sequences rather than the position of 12-mer peptides. This was done by calculating a complementary “residue score” as the average signal intensity of the consecutive 12-mer peptides in the toxin containing that given residue. In contrast to the calculation of a residue-specific alanine substitution effect, all available peptides spanning the residue when mapped to the protein sequence were used for determining the residue score.

Several of the toxins included in the experimental setup are homologs of each other and are therefore identical in parts of their sequences. To reduce the complexity in the data, the related sequences are aligned, so that the mentioned residues are displayed below each other. Also, such representation of the results allows visualisation of the effects of minor variations between similar toxins. The results for each protein subfamily are presented and discussed in the following sections with an emphasis on selected three-finger toxins and dendrotoxins, whereas the results for other toxins can be found in the Supplementary Fig. S8.

#### Short chain subfamily of the three-finger toxins

**Figure 4:**
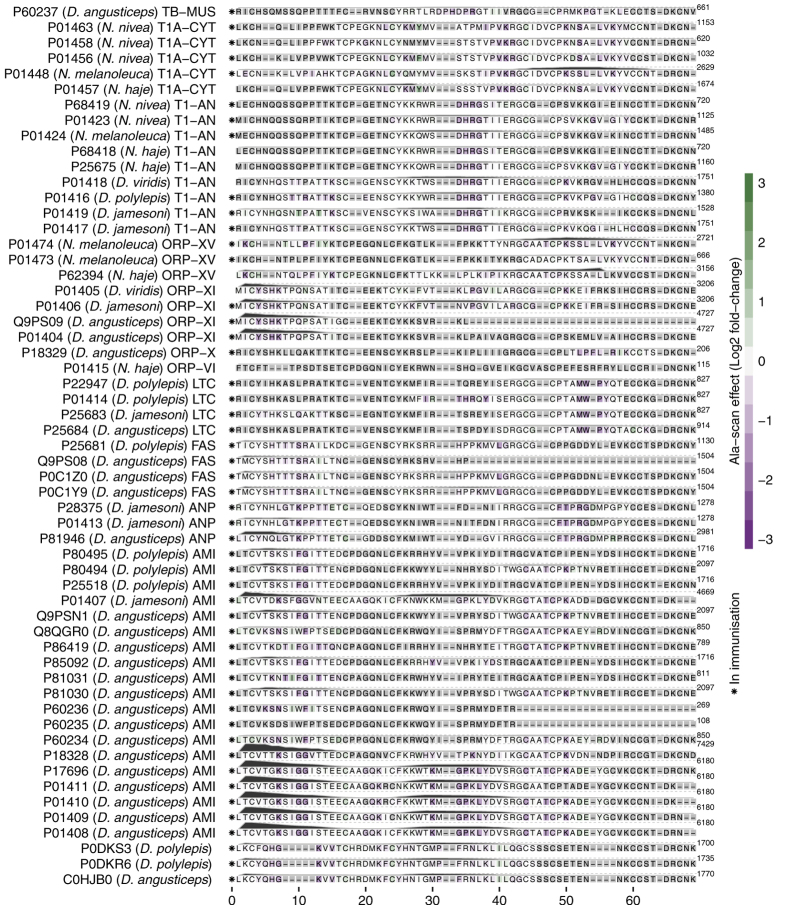
B-cell epitope analysis of the short chain subfamily of three-finger toxins recognised by the SAIMR polyvalent antivenom. The filled profiles above each sequence represent the residue scores. The tile background represents the average alanine substitution effect. The effect is displayed in log_2_ fold-change for residues present in peptides classified as epitope-containing peptides (see text for details). When no 12-mer peptide covering a given residue passed the epitope-threshold, the residue is coloured gray. Dark purple indicates that a residue is of particular importance for antibody recognition. The abbreviations after each toxin entry refers to the protein sub-subfamilies as given in Tables 2 and 3.

The study includes 55 members of the short chain subfamily belonging to 11 different protein sub-subfamilies. The identified key motifs for antibody-binding were found to be protein sub-subfamily specific with epitopes across all three loops and the N-terminal of the short chain three-finger toxins (Fig. 4).

**Figure 5:**
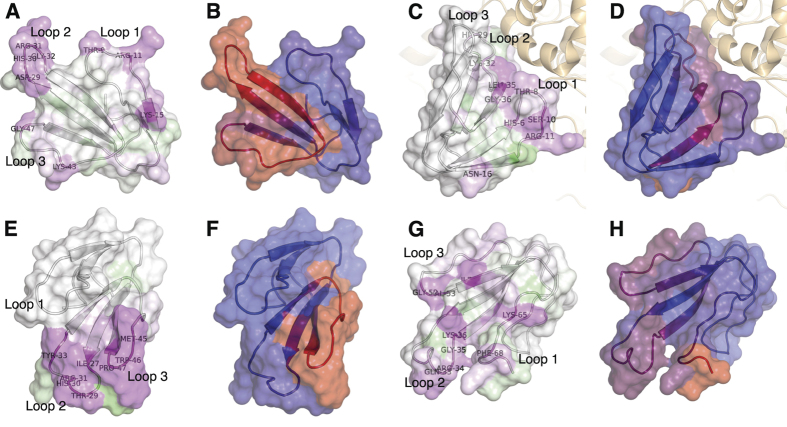
Structural presentation of B-cell epitope analysis. **(A,B)** Short neurotoxin 1 (P01416) from *D. polylepis* as an example of a type 1 α-neurotoxin. Structure built upon^54^; **(C,D)** Fasciculin-2 (P0C1Z0) from *D. angusticeps* as an example of a fasciculin. The Fasciculin-2 is co-crystallised with the human acetylcholinesterase enzyme. Structure built upon^55^; **(E,F)** Toxin FS-2 (P01414) from *D. polylepis* as an example of an L-type calcium channel blocker. Structure built upon^35^; **(G,H)** Alfa-elapitoxin-Dpp2c (P01397) from *D. polylepis* as an example of a type 2 α-neurotoxin. Structure built upon^56^. **(A,C,E,G)** Residues coloured according to alanine substitution effect in log_2_ fold-change, where magenta indicates that a residue is of particular importance for antibody recognition. Residue numbers refer to original sequence and not alignment; **(B,D,F,H)** Residues coloured according to residue score, where dark red refers to resides with high residue score, and blue refers to residues with low residue scores.

One of the most important toxin groups contributing to the high lethality of the venoms of many elapids, including *D. polylepis*, is the short chain (type 1) α-neurotoxins^22^, of which four originate from mamba and five from cobra species. For this group of α-neurotoxins, the most noticeable residue scores and effects of substitution for alanine were observed for the motif DHRG found in the alignment from position 34 to 37. All the residues in DHRG are found in loop 2 of the three-finger structure (Fig. 5A,B), wherein Arg^36^ is known to be particularly crucial for the interaction between α-neurotoxins and their targets, the neuromuscular nicotinic acetylcholine receptors (nAChRs)^27^,^32^. Furthermore, it is noteworthy that an area covering loop 1 also seems to be recognised by the antivenom antibodies for all four mamba toxins in this sub-subfamily, although the residue scores in this region are lower than for the DHRG motif. This area has been reported to be important for the interaction with carbohydrate moieties (glycosylations) on the nAChR^32^. The charged and polar residues in the type 1 α-neurotoxins, including Lys^15^, seem to be important for the recognition of mamba toxin epitopes (see Figs 4 and 5). Even though Lys^15^ is also found in the five similar cobra toxins, the variation in the surrounding residues is likely to cause their observed lack of antibody binding. The most notable differences between this region of the mamba and cobra type 1 α-neurotoxins is Pro^12^ and Pro^18^ in the cobra toxins, which are respectively replaced with alanine or simply excised in the mamba toxins. These differences might explain why the SAIMR antivenom has a preference for loop 1 in mamba type 1 α-neurotoxins over the equivalent cobra toxins. The therapeutic relevance of this difference may, however, be limited if antibody binding to the DHRG motif is sufficient to neutralise the toxic effects of all the toxins in this protein sub-subfamily.

The SAIMR antivenom is produced using venoms, which contain six of the nine type 1 α-neurotoxins included in this study (see markers in Fig. 4 left of each sequence). However, the three toxins from the venoms of *D. viridis* and *N. haje* were also found to bind the antivenom antibodies via parts of their sequences shared with the toxins included in the immunisation. This indicates the possible presence of para-specificity for the SAIMR antivenom towards the investigated *D. viridis* and *N. haje* type 1 α-neurotoxins, despite the absence of venoms from these species in the immunisation mixture.

Fasciculins are another medically important subfamily of short chain three-finger toxins found primarily in *D. angusticeps* venom^21^,^23^. As venom from *D. angusticeps* is included in the immunisation mixture for the SAIMR antivenom, the detected epitopes are likely to be a result of specific antibodies. From co-crystallisation of fasciculin-2 (P0C1Z0) with the AChE enzyme, the binding site of fasciculin-2 to the enzyme has been found to mainly include the residues from positions 6–12 (in loop 1) and 27–37 (in loop 2)^33^. In this study, we find high residue scores for residues in exactly these two parts of the toxins. Using the crystal structure of fasciculin-2 for mapping of residue scores and alanine substitution effects reveals the presence of a connected structural epitope as the residues His^6^ and Thr^8^ are found in close proximity to the Leu^35^ and Gly^36^ in the protein model in Fig. 5C,D.

The experimental setup included four members of the L-type calcium channel blocker subfamily of toxins, all originating from mamba snakes. Figure 4 shows that the SAIMR antivenom recognises the longest fully conserved region between these four toxins, covering residues from Arg^42^ to Thr^58^. The residues between Pro^49^ and Gln^57^ (Met^52^ to Tyr^56^ in particular) are found to be critical for toxin and antivenom interaction. It is of particular interest that the identified binding motif MWPY in position 52 to 56 is previously described to be the Ca^2+^ channel binding site of this toxin group^34^.

The alanine substitution effect and the residue scores are presented in three dimensions in Fig. 5E,F, exploiting the structure of Toxin FS-2 (P01414) for which an experimental NMR structure is available^35^. From this rendering, it is revealed that the two distinct antibody-binding areas found on the primary sequence of the toxin are located in two loop structures situated close to each other. It is therefore possible that these represent one epitope containing two discontinuous linear elements where one of them is barely detectable and mainly revealed in the alanine substitution analysis (Fig. 5E,F). Still, this hypothesis has yet to be validated experimentally.

The examples above with the toxin families known to play major roles in toxicity, illustrate how antibody recognition of each toxin sub-subfamily can be explored in detail. The results for 40 additional short chain three-fingered toxins are available in Fig. 4, including 19 aminergic (muscarinic) toxins known to act synergistically^21^. However, a further exploration of these antivenom-toxin interactions is beyond the scope of this communication.

#### Long chain neurotoxins of the three-finger toxin family

**Figure 6:**
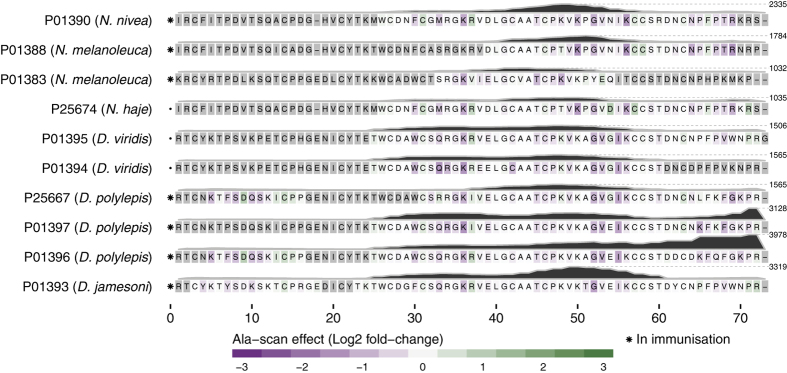
Linear B-cell epitope analysis of ten long chain subfamily three-finger toxins recognised by the SAIMR antivenom. See Fig. 4 and text for details.

All ten long chain three-finger toxins included in the study are type 2 α-neurotoxins. Similar to type 1 α-neurotoxins, type 2 α-neurotoxins are inhibitors of neuromuscular nAChRs^32^. Six of the ten type 2 α-neurotoxins originate from mamba species and four from cobra species. The effect of alanine substitutions and residue scores were mapped to the aligned protein sequences, illustrated in Fig. 6.

The majority of the residues important for antibody-toxin binding are located in loop 2 (position 22 to 42 in the alignment), loop 3 (position 47 to 58), and in the C-terminal. This pattern surprisingly differentiates itself from the binding pattern observed for type 1 α-neurotoxins (Fig. 4), where linear parts of epitopes were identified in loop 1 and 2. The key residues found in the alanine scans are scattered over a large section of the toxins with no short distinctive motifs, although residues in position 30 to 36 and 45 to 56 seem to be slightly more important for antibody recognition for most long chain α-neurotoxins. The observed binding across two large toxin segments could potentially indicate that a mixture of antibodies binding to overlapping epitopes are present in the SAIMR antivenom, given that the binding region of an antibody is usually limited to around nine residues^30^. Nevertheless, a number of residues including Gln^33^, Lys^36^, Gly^52^, Val^53^, and Ile^54^ seem important in most of the epitopes. The spatial distribution of the most important residues is illustrated in Fig. 5G,H.

It is noteworthy that although type 1 and type 2 α-neurotoxins target the same receptor, no similar epitope is identified. We therefore hypothesise that no or very little antibody cross-recognition takes place between the two types of α-neurotoxins. This implies that even though the clinical manifestations of a snakebite might include flaccid paralysis from abrogation of neuromuscular transmission due to the effects of α-neurotoxins, it is crucial for an efficacious antivenom to recognise and neutralise the specific subtype of α-neurotoxins present in the venom of the snake species responsible for the bite.

The residues of type 2 α-neurotoxins involved in nAChR inhibition have been investigated for a limited number of toxins, including α-bungarotoxin from *Bungarus multicinctus*^36^,^37^ and α-elapitoxin-Nno2a from *Naja naja oxiana*^38^. These studies uncovered interactions between the neuromuscular nAChR and all three loops of the three-finger structure of the toxins, thus involving a high number of interacting residues. For α-bungarotoxin, 34 out of 74 residues were involved in binding. All but one of the residues found to be important for antibody recognition in the mamba toxins corresponded to the very same residues found to be important in α-bungarotoxin interaction with the nAChR. The only exception is Gly^52^, which was not part of the interaction for α-bungarotoxin although it is located in loop 2 (Fig. 5G,H). However, this small amino acid may be important for conformational flexibility required to position the loops appropriately for antibody (and perhaps also receptor) binding for the mamba toxins.

For most type 2 α-neurotoxins, binding between the SAIMR antivenom and two or more non-overlapping 12-mers classified as antibody binders was observed, even though three of the toxins originate from snake species not included in the SAIMR immunisation mixture. This para-specificity can be explained by the presence of completely conserved sections between these three toxins and the mamba and cobra toxins included in the immunisation mixture. On the other hand, for sub-sequences, where amino acids are substituted, para-specificity can easily be lost. This is illustrated by the C-terminal of α-elapitoxin-Dv2b (P01394) from *D. viridis*, which is not recognised by the antivenom at all (see Fig. 6), although only two amino acids (Asp^64^, Lys^69^) are altered compared to α-elapitoxin-Dv2a (P01395), also from *D. viridis*. This example illustrates that high similarity is not sufficient to predict antibody binding if the key residues for the interaction are not known.

#### Dendrotoxins of Kunitz-type inhibitor family

**Figure 7:**
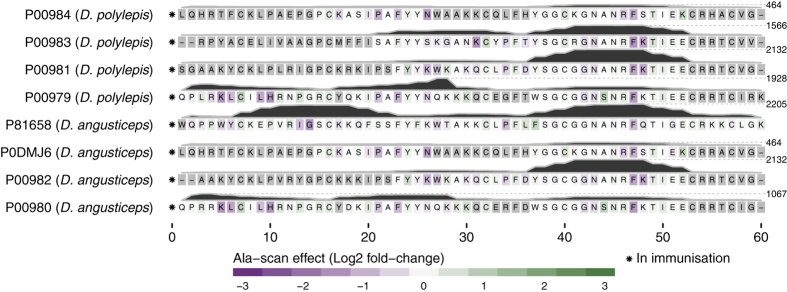
Linear B-cell epitope analysis of eight dendrotoxins recognised by the SAIMR antivenom. See Fig. 4 and text for details.

The study included eight dendrotoxins from *D. polylepis* and *D. angusticeps* (Fig. 7). In each case, at least three overlapping antibody-binding 12-mer peptides were identified in position 37 to 52, in which particularly Phe^47^ was found to be crucial for antibody recognition in all cases. Based on residue scores observed in this region, the eight dendrotoxins can be divided in two distinct groups. The high scoring toxins differed from the low scoring toxins in position 44 and 48, where Ala^44^ and Lys^48^/Gln^48^ (high scoring toxins) were replaced with a serine in one of the positions. The four dendrotoxins showing decreased antibody-binding for residues between position 37 to 52 do, however, all display a weak but shared epitope PAFYYN in alignment position 21 to 26. The Pro^21^, Phe^23^, and Asn^26^ seem particularly important in the alanine substitution analysis, although the replacement of Ala^22^ with serine completely abrogates antibody binding to the epitope. This variability within dendrotoxins containing Ala or Ser in similar positions (Ala and Ser codons are only one base pair apart) is likely to have major consequences in terms of immunogenicity and cross-recognition of the raised antibodies in an antivenom. This may explain the previously observed absence of significant binding in an ELISA experiments to one of the dendrotoxin-containing venom fractions from *D. polylepis*, where the very same antivenom was employed^21^.

### Antivenoms tend to bind to functional sites of toxins

The relative number of epitopes containing sufficiently large linear elements to be identified in this peptide microarray setup is unknown and not addressed here. Therefore, it is theoretically possible that the corresponding antibodies are not those that protect a victim from the consequences of snakebite envenoming. However, the binding data presented above highlights an intriguing finding. For most of the toxins, the epitopes tend to coincide with the site involved in toxin function. A similar finding was recently reported for the binding of camelid single-domain antibody fragments to two phospholipases A_2_ from *Bothrops jararacussu* venom^39^. From an evolutionary perspective, toxins have evolved to subdue prey and deter predators by specifically targeting essential receptors and enzymes, while remaining inert in terms of molecular interactions with other biomolecules^3^,^27^,^40^. Thus, the site involved in toxin function contains a distinctive motif capable of engaging in strong interactions based on supramolecular recognition, whereas the rest of the toxin molecule may have evolved to engage in as few molecular interactions as possible. On speculative grounds, it may be conceived that the emergence of ‘toxic motifs’ from the conserved scaffolds of proteins having normal physiological roles in snakes implies evolutionary structural novelty. Therefore, novel functional sites in the proteins recruited in venoms to become toxins may not commonly find close homologs in immunised animals, which would possibly focus the adaptive humoral response of the animals toward such functional sites. Possibly, the distinctive motifs of the functional sites of toxins not only allow high affinity interactions to take place between toxin and target, but also between antivenom antibodies and toxins, although it is very likely that any small neurotoxin (7–9 kDa in size) would theoretically be neutralised regardless of binding site when encountered by a much larger F(ab′)_2_ (100 kDa) or IgG (150 kDa). Given that the investigated antivenoms have been shown to recognise and neutralise mamba toxins^21^,^22^, the recognition of epitopes at the functional site of the toxins may explain the molecular basis for toxin neutralisation through abrogation of toxin-target interaction. This elucidation of epitope-paratope couples may be employed to advance our understanding of how the immune systems of production animals select and target antigens during immunisation. In turn, this knowledge can be used to optimise and balance serum-based antivenoms through engineering of immunisation mixtures to enhance the antibody response against weakly immunogenic, yet medically important toxins^41^, such as dendrotoxins^22^.

## Conclusion

With this study, high-density peptide microarray technology is for the first time exploited for identification of linear epitope elements in snake venom toxins. The results obtained clearly demonstrate the power of this high-throughput approach compared to similar methods based on SPOT synthesis^6^,^7^,^12^ and mutation analysis^11^. Furthermore, toxin epitopes recognised by antivenom antibodies were unveiled for all but two of the manually curated toxins from every mamba and four cobra species endemic to sub-Saharan Africa. These findings also point towards the existence of a correlation between the active/functional site of snake venom neurotoxins and the site recognised by antivenom antibodies.

The potential of harnessing high-density peptide microarrays for identification of important toxin epitopes involved in toxin function and neutralisation by antivenoms may be utilised for design of next generation antivenoms based on DNA immunisation and immunisation with synthetic epitope strings^13^. DNA immunisation has been explored by a number of research groups^42^,^43^,^44^,^45^,^46^,^47^, as has the use of synthetic epitope strings in the field of spider and scorpion antivenoms^48^. The possible benefits of using DNA immunisation methods or synthetic epitope strings for immunisation include the independence of snake venoms and the ability to select only the medically important toxins for the immunisation process. Snake venoms are both expensive and time consuming to procure, and they may be a source for antivenom batch-to-batch variation due to differences in venom composition for individual snakes^49^. Therefore, identification of key epitopes for medically important snake toxins may provide a new avenue towards less expensive antivenoms based on DNA immunisation or synthetic epitopes strings with better therapeutic value due to a well-balanced antibody response against important venom components.

Finally, the immuno-profiling approach presented here may represent a novel method for assessing para-specificity of both antivenoms, individual antibodies, and oligoclonal antibody mixtures. High-density peptide microarrays could thus potentially be used as an indicative high-throughput predictor of what venoms and toxins a given antivenom (or antibody) may be able to neutralise.

## Methods

### High-density peptide microarray design

A peptide library was generated *in silico* for synthesis on high-density peptide microarrays. The library consisted of overlapping 12-mers representing the primary sequence of the snake neurotoxins listed in Tables 2 and 3 tiled at every second amino acid. All (562) redundant (non-unique) peptides were removed and the remaining 1,588 peptides were replicated five times. Furthermore, the *in silico* library was expanded to include peptides representing all possible point mutations to alanine, generated from each unique peptide. The 18,468 unique alanine substitution peptides were included in one replicate resulting in a library of 26,408 peptides in total. Finally, the individual peptides in the library were assigned random positions on the microarray in order to minimise the impact of local intensity biases.

### High-density peptide microarray synthesis

Six identical microarrays were synthesised with a Roche-NimbleGen Maskless Array Synthesiser (MAS) by light-directed solid-phase peptide synthesis using an amino-functionalised surface coupled with a 6-aminohexanoic acid linker and amino acid derivatives carrying a photosensitive 2-(2-nitrophenyl)propyloxycarbonyl (NPPOC) protection group. Amino acid coupling was performed in dimethylformamide (DMF) for 3 minutes using amino acids pre-activated with HBTU as an activator, hydroxybenzotriazole (HOBt) to suppress racemisation, and *N*,*N*-diisopropylethylamine as base. Following each coupling step, the microarray was washed with *N*-methyl-2-pyrrolidone (NMP), and site-specific cleavage of the NPPOC protection group was accomplished by irradiation of an image created by a Digital Micro-Mirror Device (Texas Instruments, SXGA+graphics format), projecting 365 nm wavelength light. Coupling cycles were repeated to synthesise the full *in silico* generated peptide library. Final removal of side-chain protection groups was performed with 95% trifluoroacetic acid/4.5% water/0.5% triisopropylsilane for 30 minutes.

### Antivenoms

Three polyspecific antivenoms were investigated: (a) SAIMR (South African Institute for Medical Research) Polyvalent Snake Antivenom from South African Vaccine Producers (Pty) Ltd. (batch number BC02645, expiry date 07/2016); (b) Snake Venom Antivenom (Central Africa) from VINS Bioproducts Ltd. (batch 12AS13002, expiry date 04/2017); and (c) Snake Venom Antivenom (African) from VINS Bioproducts Ltd. (batch 13022, expiry date 01/2018). All of them are of equine origin and consist of F(ab′)_2_ fragments obtained by pepsin digestion of antibodies. The reported immunisation mixtures of the horses are summarised in Table 1. Protein concentrations for the antivenoms were measured by their absorbance at 280 nm on a NanoDrop 2000c instrument, Thermo Scientific.

### Sample binding and processing

The microarrays were incubated overnight at 4 °C with individual antivenom samples mixed with binding buffer at either 1:50 or 1:100 dilution to a final volume of 100 *μ*L. This was followed by three 10 minute washes with a TBST (Tris Buffered Saline and Tween 20) buffer and incubating with goat anti-horse IgG (H+ L) conjugated with Alexa Flour^®^ 647 (Jackson ImmunoResearch, 108-605-003) at room temperature for 3 hours. After a final wash, the arrays were dried and read using an MS200 microarray scanner, and signals were extracted using NimbleGen DEVA signal extraction software

### Clasification of peptides as antibody-binders

The 1,588 unique peptides derived from the original toxin sequences (the non-alanine substituted) were each present in five replicates in each microarray. The median signal intensities were determined and used to classify peptides as either antibody-binding or non-antibody-binding. This classification was performed on the basis of the lower 70-percentile of the median signals, as this subset of peptides were assumed to originate from unspecific binding. The lower 70-percentile was chosen over other (more or less conservative) percentiles as the resulting distributions of signals closely resembled normal distributions (data not shown). For each microarray experiment, the mean and the standard deviation of the subsets representing non-specific binding was determined (Supplemental Table S1) and a peptide was classified as an antibody target if the median signal intensity was more than 10 standard deviations above the mean.

Venn diagrams showing the overlap of the peptides classified as antibody-binders were constructed using R package VennDiagram^50^.

### Alanine substitution analysis

The alanine substitution analysis was performed using two custom-built Perl programs. The first program went through all possible 12-mer peptides derived from an input fasta file. The signal intensities of each peptide were stored as arrays in a hash structure with the peptide sequences serving as keys. When a peptide was found to have a median signal intensity above the experiment-specific threshold defined above, the signal intensities of the alanine substituted peptides were located in the hash structure. For each peptide, an array containing the relative effect of replacing each position for alanine was built and written to an output file used for the second custom-built program. For non-antibody-binding peptides, the relative alanine substitution effect was not assigned.

The second program was designed to loop over each protein entry and add the generated alanine substitution data and median signal data to two separate arrays, where each array element represents an amino acid residue in the original protein sequence. Using two other arrays to count the number of times data was added to each residue, the average alanine substitution effect of each residue and the average median signal of peptides containing a given residue (referred to as residue score) were determined. The resulting scores were written to an output tab-separated file, which was imported in R for data visualisation using the ggplot2 package^51^.

All the programs built for this study were prepared to handle gap characters (−). This feature enables the alignment of protein sequences in the input fasta file and generation of figures, such as Fig. 4. Here, Clustal Omega was used for multiple sequence alignment^52^.

### Protein modeling

The residue scores and the average alanine substitution effect of each residue were mapped to available protein structures by modifying the temperature factors on the relevant pdb files using the R package Rpdb^53^. All images were generated using an open source built version of The PyMOL Molecular Graphics System.

## Additional Information

**How to cite this article**: Engmark, M. *et al*. High-throughput immuno-profiling of mamba (*Dendroaspis*) venom toxin epitopes using high-density peptide microarrays. *Sci. Rep.* **6**, 36629; doi: 10.1038/srep36629 (2016).

**Publisher’s note:** Springer Nature remains neutral with regard to jurisdictional claims in published maps and institutional affiliations.

## Supplementary Material

Supplementary Information

Supplementary Dataset 1

Supplementary Dataset 2

Supplementary Dataset 3

Supplementary Dataset 4

Supplementary Dataset 5

Supplementary Dataset 6

## Figures and Tables

**Table 1 t1:** Immunisation mixtures for antivenoms used in the study, specified by their manufacturers.

Antivenom	Manufacturer	Venoms in immunisation	Protein concentration
SAIMR PolyvalentSnake Antivenom	South AfricanVaccine Producers(Pty) Ltd	*Dendroaspis angusticeps*	171.6 mg/mL
		*D. jamesoni*	
		*D. polylepis*	
		*Naja annulifera*	
		*N. melanoleuca*	
		*N. mossambica*	
		*N. nivea*	
		*Bitis arietans*	
		*B. gabonica*	
		*Haemachatus haemachatus*	
Snake VenomAntiserum(African)	VINS Bioproducts	*D. jamesoni*	64.4 mg/mL
		*D. polylepis*	
		*D. viridis*	
		*N. haje*	
		*N. melanoleuca*	
		*N. nigricollis*	
		*B. arietans*	
		*B. gabonica*	
		*Echis leucogaster*	
		*E. ocellatus*	
Snake VenomAntiserum(Central Africa)	VINS Bioproducts	*D. polylepis*	90.5 mg/mL
		*B. gabonica rhinoceros*	
		*Vipera (Daboia) russelli*	
		*E. carinatus*	

**Table 2:** Toxins included in the study part 1.

Snake species	UniProt entry	Length	Protein subfamily	Protein sub-subfamily
*D. polylepis*	P25687	81	AVIT (prokineticin)	
*D. angusticeps*	P28374	38	Natriuretic peptide	
*D. angusticeps*	Q8QGP7	53	Natriuretic peptide	
*N. melanoleuca*	P01383	71	Long-chain 3FTx	T2-α-NT
*N. melanoleuca*	P01388	71	Long-chain 3FTx	T2-α-NT
*N. nivea*	P01390	71	Long-chain 3FTx	T2-α-NT
*N. haje*	P25674	71	Long-chain 3FTx	T2-α-NT
*D. jamesoni*	P01393	72	Long-chain 3FTx	T2-α-NT
*D. viridis*	P01394	72	Long-chain 3FTx	T2-α-NT
*D. viridis*	P01395	73	Long-chain 3FTx	T2-α-NT
*D. polylepis*	P01396	72	Long-chain 3FTx	T2-α-NT
*D. polylepis*	P01397	72	Long-chain 3FTx	T2-α-NT
*D. polylepis*	P25667	72	Long-chain 3FTx	T2-α-NT
*N. melanoleuca*	P01400	65	Non-conventional 3TFx	ORP-II
*N. haje*	P01401	65	Non-conventional 3TFx	ORP-II
*N. nivea*	P25680	65	Non-conventional 3TFx	ORP-II
*D. jamesoni*	P25682	63	Non-conventional 3TFx	ORP-XIX
*D. angusticeps*	C0HJB0	57	Short-chain 3FTx	
*D. polylepis*	P0DKR6	78	Short-chain 3FTx	
*D. polylepis*	P0DKS3	57	Short-chain 3FTx	
*D. angusticeps*	P0C1Y9	61	Short-chain 3FTx	FAS
*D. angusticeps*	P0C1Z0	61	Short-chain 3FTx	FAS
*D. polylepis*	P25681	61	Short-chain 3FTx	FAS
*D. angusticeps*	Q9PS08	30*	Short-chain 3FTx	FAS
*D. jamesoni*	P01407	62	Short-chain 3FTx	AMI
*D. angusticeps*	P01408	63	Short-chain 3FTx	AMI
*D. angusticeps*	P01409	63	Short-chain 3FTx	AMI
*D. angusticeps*	P01410	62	Short-chain 3FTx	AMI
*D. angusticeps*	P01411	62	Short-chain 3FTx	AMI
*D. angusticeps*	P17696	86	Short-chain 3FTx	AMI
*D. angusticeps*	P18328	86	Short-chain 3FTx	AMI
*D. polylepis*	P25518	65	Short-chain 3FTx	AMI
*D. angusticeps*	P60234	65	Short-chain 3FTx	AMI
*D. angusticeps*	P60235	40*	Short-chain 3FTx	AMI
*D. angusticeps*	P60236	40*	Short-chain 3FTx	AMI
*D. polylepis*	P80494	66	Short-chain 3FTx	AMI
*D. polylepis*	P80495	65	Short-chain 3FTx	AMI
*D. angusticeps*	P81030	66	Short-chain 3FTx	AMI
*D. angusticeps*	P81031	65	Short-chain 3FTx	AMI
*D. angusticeps*	P85092	65	Short-chain 3FTx	AMI
*D. angusticeps*	P86419	66	Short-chain 3FTx	AMI
*D. angusticeps*	Q8QGR0	86	Short-chain 3FTx	AMI
*D. angusticeps*	Q9PSN1	66	Short-chain 3FTx	AMI

Asterisk marks when an entry represents a fragment of the full sequence. Abbreviations: 3FTx (Three-finger toxin), T2-α-NT (Type 2 α-neurotoxin), ORP-II (orphan group II toxin), ORP-XIX (orphan group XIX toxin), FAS (Fasciculin), AMI (Aminergic toxin).

**Table 3:** Toxins included in the study part 2.

Snake species	UniProt entry	Length	Protein subfamily	Protein sub-subfamily
*D. jamesoni*	P01413	61	Short-chain 3FTx	ANP
*D. jamesoni*	P28375	59	Short-chain 3FTx	ANP
*D. angusticeps*	P81946	59	Short-chain 3FTx	ANP
*D. polylepis*	P01414	60	Short-chain 3FTx	LTC
*D. polylepis*	P22947	60	Short-chain 3FTx	LTC
*D. jamesoni*	P25683	60	Short-chain 3FTx	LTC
*D. angusticeps*	P25684	60	Short-chain 3FTx	LTC
*N. haje*	P01415	61	Short-chain 3FTx	ORP-VI
*D. angusticeps*	P18329	80	Short-chain 3FTx	ORP-XI
*D. angusticeps*	P01404	81	Short-chain 3FTx	ORP-XI
*D. viridis*	P01405	60	Short-chain 3FTx	ORP-XI
*D. jamesoni*	P01406	60	Short-chain 3FTx	ORP-XI
*D. angusticeps*	Q9PS09	30*	Short-chain 3FTx	ORP-XI
*N. melanoleuca*	P01473	61	Short-chain 3FTx	ORP-XV
*N. melanoleuca*	P01474	61	Short-chain 3FTx	ORP-XV
*N. haje*	P62394	62	Short-chain 3FTx	ORP-XV
*D. angusticeps*	P60237	63	Short-chain 3FTx	TB-MUS
*N. nivea*	P01423	61	Short-chain 3FTx	T1-α-NT
*N. melanoleuca*	P01424	61	Short-chain 3FTx	T1-α-NT
*N. haje*	P25675	61	Short-chain 3FTx	T1-α-NT
*N. haje*	P68418	61	Short-chain 3FTx	T1-α-NT
*N. nivea*	P68419	61	Short-chain 3FTx	T1-α-NT
*D. polylepis*	P01416	60	Short-chain 3FTx	T1-α-NT
*D. jamesoni*	P01417	60	Short-chain 3FTx	T1-α-NT
*D. viridis*	P01418	60	Short-chain 3FTx	T1-α-NT
*D. jamesoni*	P01419	58	Short-chain 3FTx	T1-α-NT
*N. melanoleuca*	P01448	60	Short-chain 3FTx	T1A-CYT
*N. nivea*	P01456	60	Short-chain 3FTx	T1A-CYT
*N. haje*	P01457	60	Short-chain 3FTx	T1A-CYT
*N. nivea*	P01458	60	Short-chain 3FTx	T1A-CYT
*N. nivea*	P01463	60	Short-chain 3FTx	T1A-CYT
*D. polylepis*	P00979	60	Kunitz-type	
*D. angusticeps*	P00980	59	Kunitz-type	
*D. polylepis*	P00981	79	Kunitz-type	
*D. angusticeps*	P00982	57	Kunitz-type	
*D. polylepis*	P00983	57	Kunitz-type	
*D. polylepis*	P00984	59	Kunitz-type	
*D. angusticeps*	P0DMJ6	59	Kunitz-type	
*D. angusticeps*	P81658	60	Kunitz-type	

Asterisk marks when an entry represents a fragment of the full sequence. Abbreviations: 3FTx (Three-finger toxin), ANP (antiplatelet toxin), LTC (L-Type calcium channel blocker) ORP-VI (orphan group VI toxin), ORP-XI (orphan group XI toxin), TB-MUS (Type B muscarinic toxin), T1-α-NT (Type 1 α-neurotoxin), T1A-CYT (Type 1A cytotoxin).
