# Supplementary material for: High-throughput immuno-profiling of mamba (Dendroaspis) venom toxin epitopes using high-density peptide microarrays
Source: Sci Rep. 2016 Nov 8;6:36629. doi: 10.1038/srep36629 (PMC5100549; doi:10.1038/srep36629)
Supplement: Supplementary Information [file srep36629-s1.pdf]

Supplementary Information

High-throughput immuno-profiling of mamba (*Dendroaspis*) venom toxin epitopes using high-density peptide microarrays

Mikael Engmark[1,2,*], Mikael R. Andersen[2], Andreas H. Laustsen[2,3], Jigar Patel[4], Eric Sullivan[4], Federico de Masi[1], Christian S. Hansen[1], Jens V. Kringelum[1], Bruno Lomonte[5], José María Gutiérrez[5], and Ole Lund[1]

[1]Technical University of Denmark, Department of Bio and Health Informatics, Kgs. Lyngby 2800, Denmark

[2]Technical University of Denmark, Department of Biotechnology and Biomedicine, Kgs. Lyngby 2800, Denmark

[3]University of Copenhagen, Department of Drug Design and Pharmacology, Faculty of Health and Medical Sciences, Copenhagen E 2100, Denmark

[4]Roche NimbleGen, Madison, Wisconsin 53719, the United States of America

[5]Instituto Clodomiro Picado, Facultad de Microbiología, Universidad de Costa Rica, San José 11501, Costa Rica

[*]Correspondence should be addressed to ME miken@bio.dtu.dk

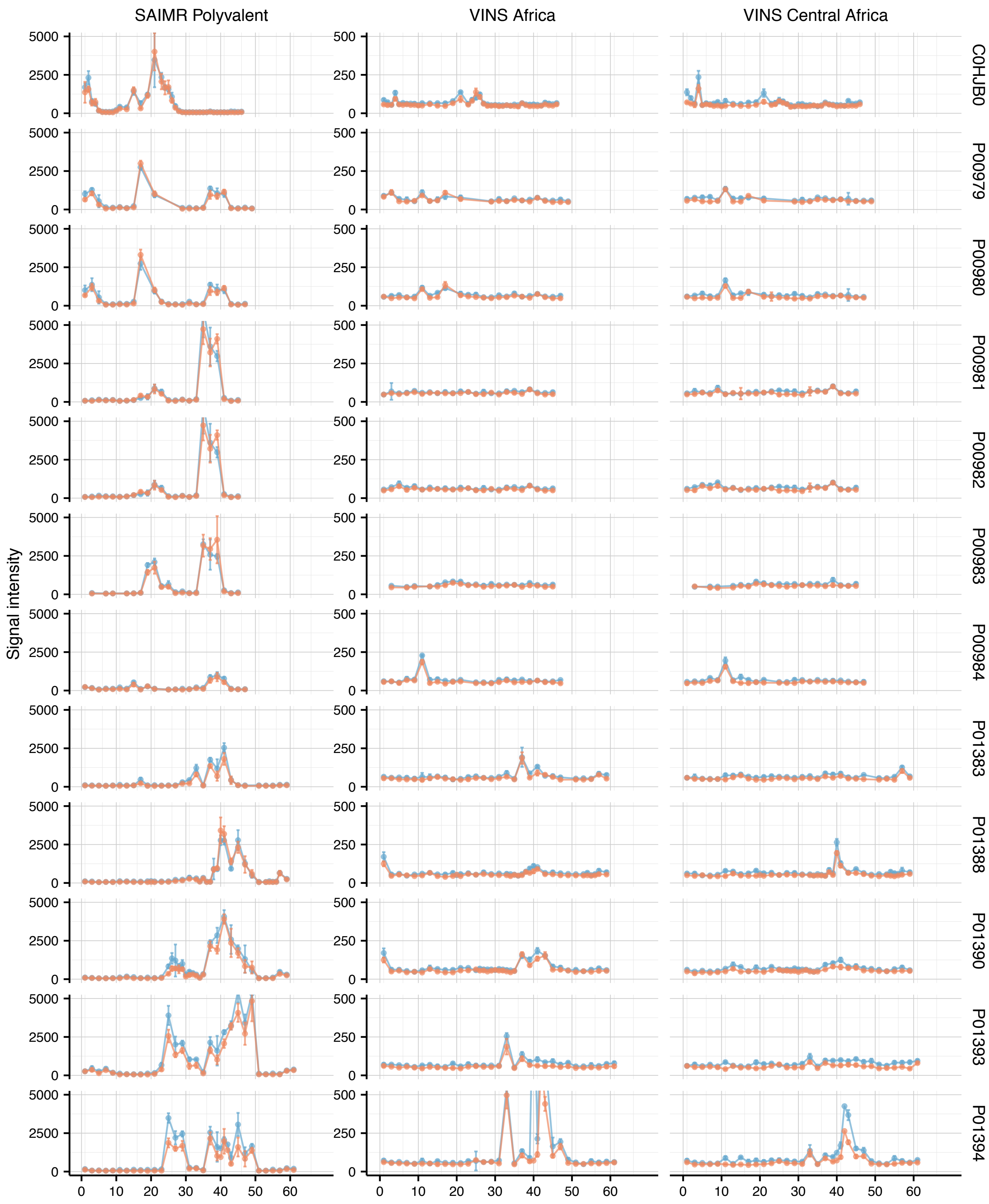

Figure S1. Binding profiles of 12 toxins. The median signal intensity of five replicates of each peptide is plotted as dots based on the position of the N-terminal residue of the 12-mer peptide in the protein sequences (pro-peptides removed). The dots are connected with straight lines to visualize the relation between overlapping peptides. The error bars represent the standard deviation of the five replicates. Positions containing gaps in the alignments were ignored. Blue dots refer to the 1:50 dilution experiment, while red dots refer to the 1:100 dilution experiments. Notice that each point contains information on the following 11 amino acids of the toxin.

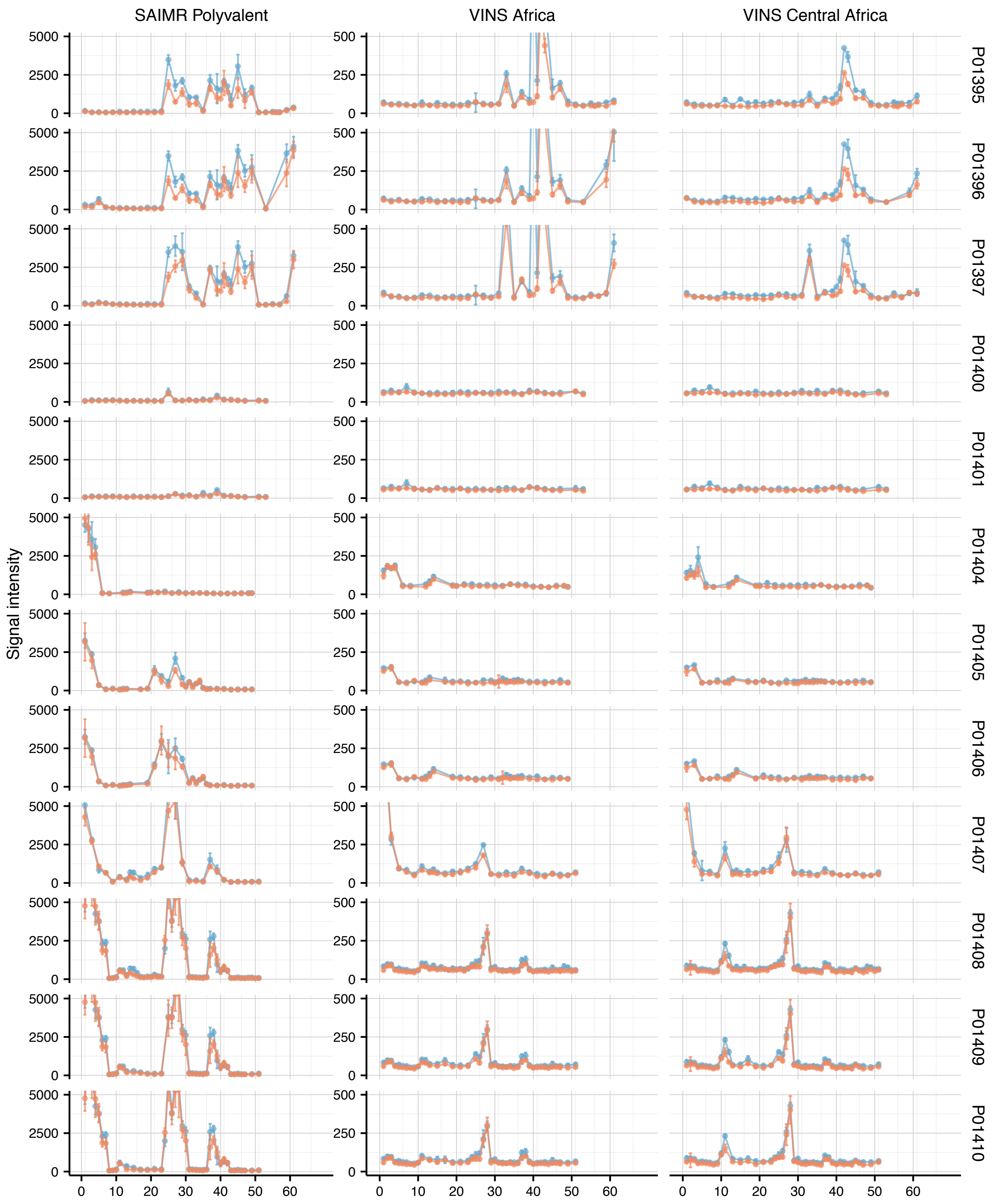

Figure S2. Binding profiles of 12 toxins. The median signal intensity of five replicates of each peptide is plotted as dots based on the position of the N-terminal residue of the 12-mer peptide in the protein sequences (pro-peptides removed). The dots are connected with straight lines to visualize the relation between overlapping peptides. The error bars represent the standard deviation of the five replicates. Positions containing gaps in the alignments were ignored. Blue dots refer to the 1:50 dilution experiment, while red dots refer to the 1:100 dilution experiments. Notice that each point contains information on the following 11 amino acids of the toxin.

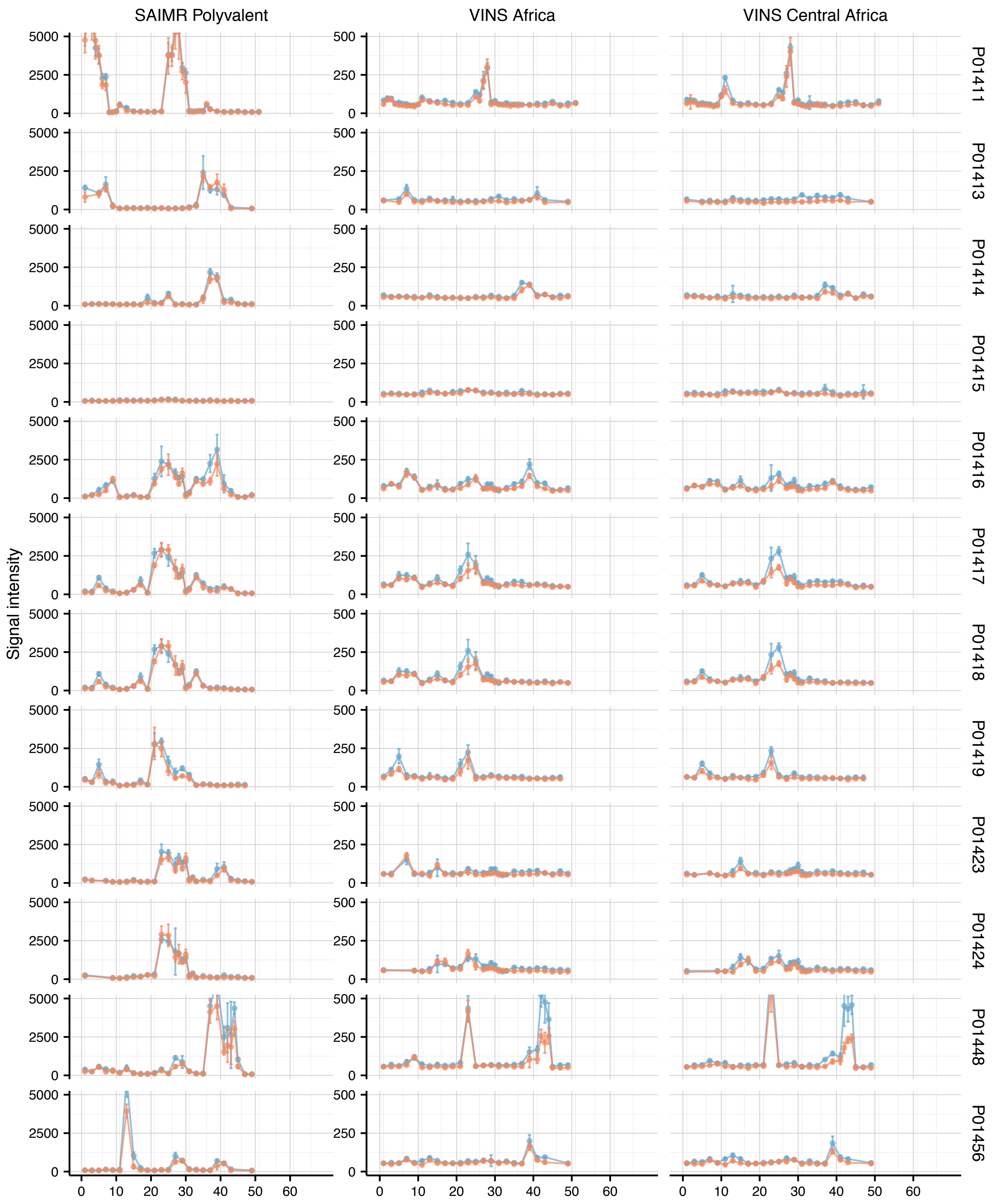

Figure S3. Binding profiles of 12 toxins. The median signal intensity of five replicates of each peptide is plotted as dots based on the position of the N-terminal residue of the 12-mer peptide in the protein sequences (pro-peptides removed). The dots are connected with straight lines to visualize the relation between overlapping peptides. The error bars represent the standard deviation of the five replicates. Positions containing gaps in the alignments were ignored. Blue dots refer to the 1:50 dilution experiment, while red dots refer to the 1:100 dilution experiments. Notice that each point contains information on the following 11 amino acids of the toxin.

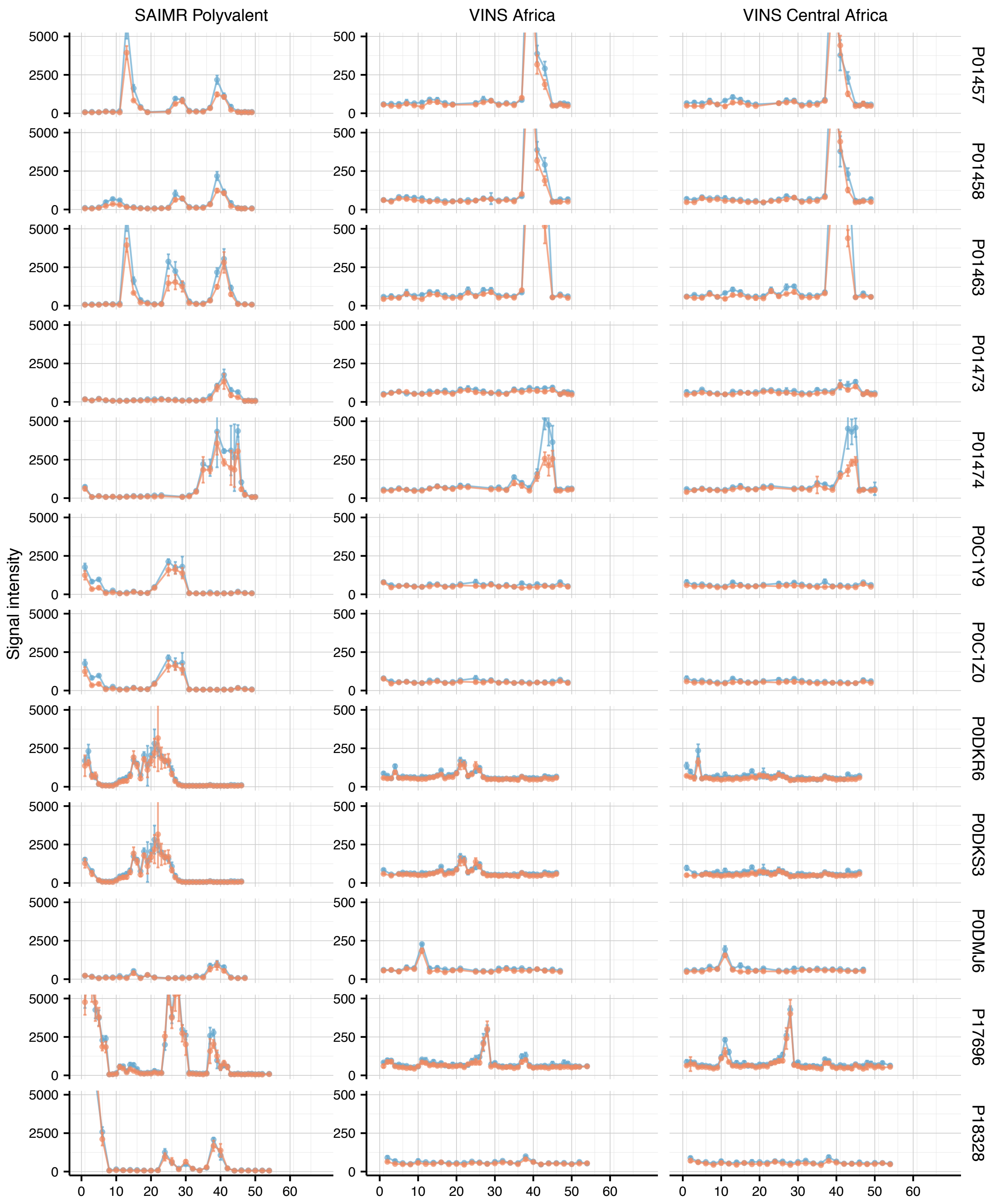

Figure S4. Binding profiles of 12 toxins. The median signal intensity of five replicates of each peptide is plotted as dots based on the position of the N-terminal residue of the 12-mer peptide in the protein sequences (pro-peptides removed). The dots are connected with straight lines to visualize the relation between overlapping peptides. The error bars represent the standard deviation of the five replicates. Positions containing gaps in the alignments were ignored. Blue dots refer to the 1:50 dilution experiment, while red dots refer to the 1:100 dilution experiments. Notice that each point contains information on the following 11 amino acids of the toxin.

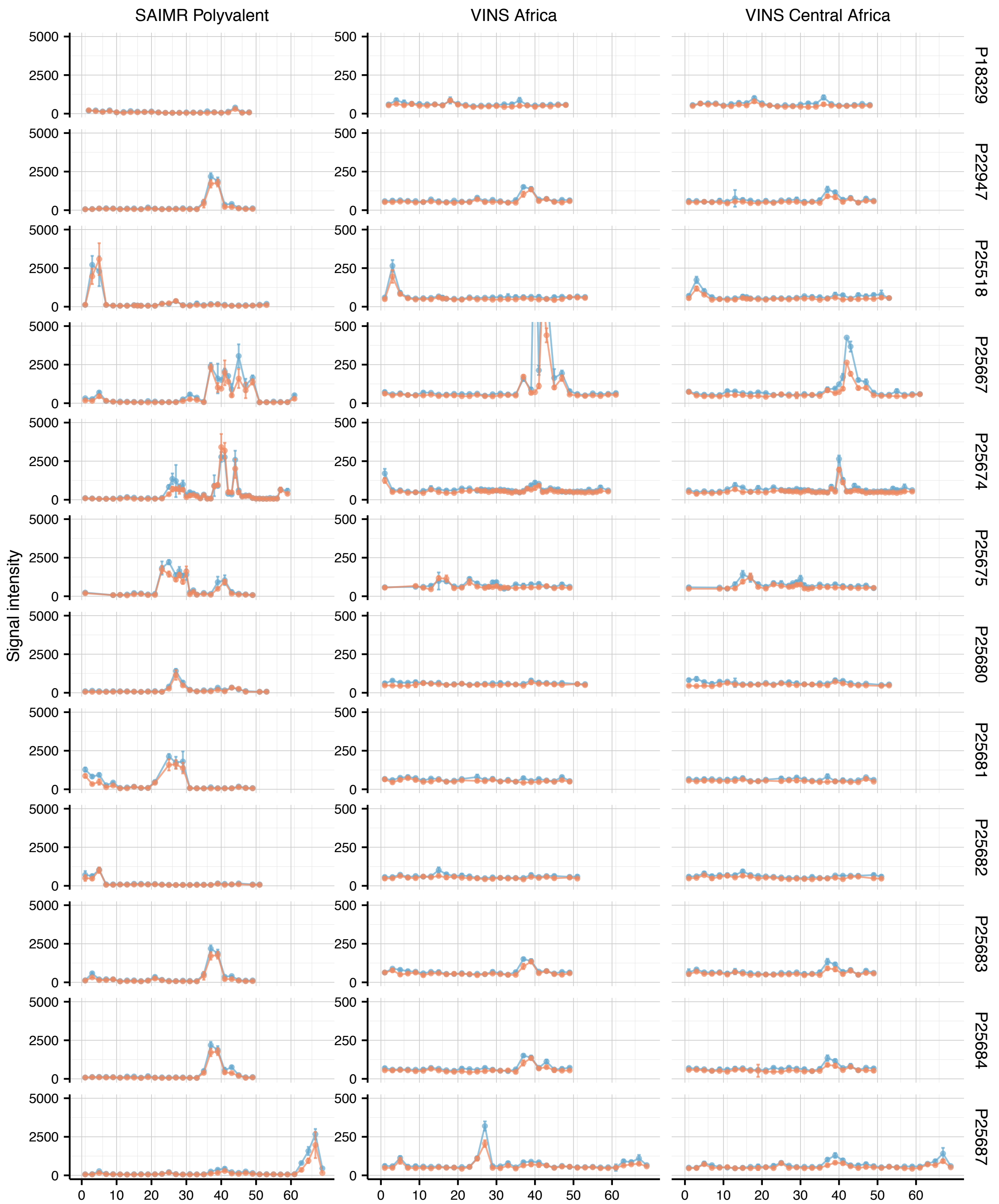

Figure S5. Binding profiles of 12 toxins. The median signal intensity of five replicates of each peptide is plotted as dots based on the position of the N-terminal residue of the 12-mer peptide in the protein sequences (pro-peptides removed). The dots are connected with straight lines to visualize the relation between overlapping peptides. The error bars represent the standard deviation of the five replicates. Positions containing gaps in the alignments were ignored. Blue dots refer to the 1:50 dilution experiment, while red dots refer to the 1:100 dilution experiments. Notice that each point contains information on the following 11 amino acids of the toxin.

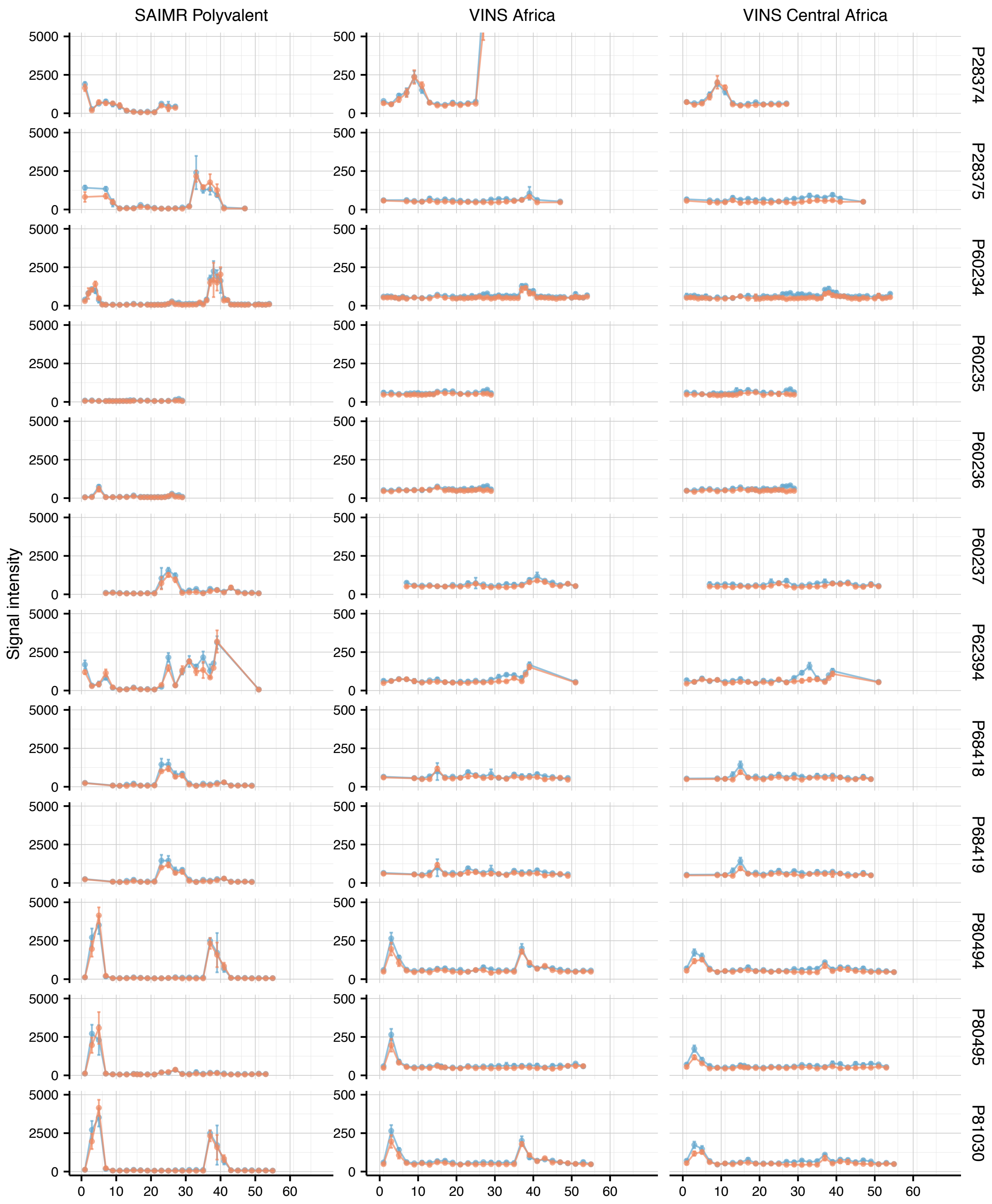

Figure S6. Binding profiles of 12 toxins. The median signal intensity of five replicates of each peptide is plotted as dots based on the position of the N-terminal residue of the 12-mer peptide in the protein sequences (pro-peptides removed). The dots are connected with straight lines to visualize the relation between overlapping peptides. The error bars represent the standard deviation of the five replicates. Positions containing gaps in the alignments were ignored. Blue dots refer to the 1:50 dilution experiment, while red dots refer to the 1:100 dilution experiments. Notice that each point contains information on the following 11 amino acids of the toxin.

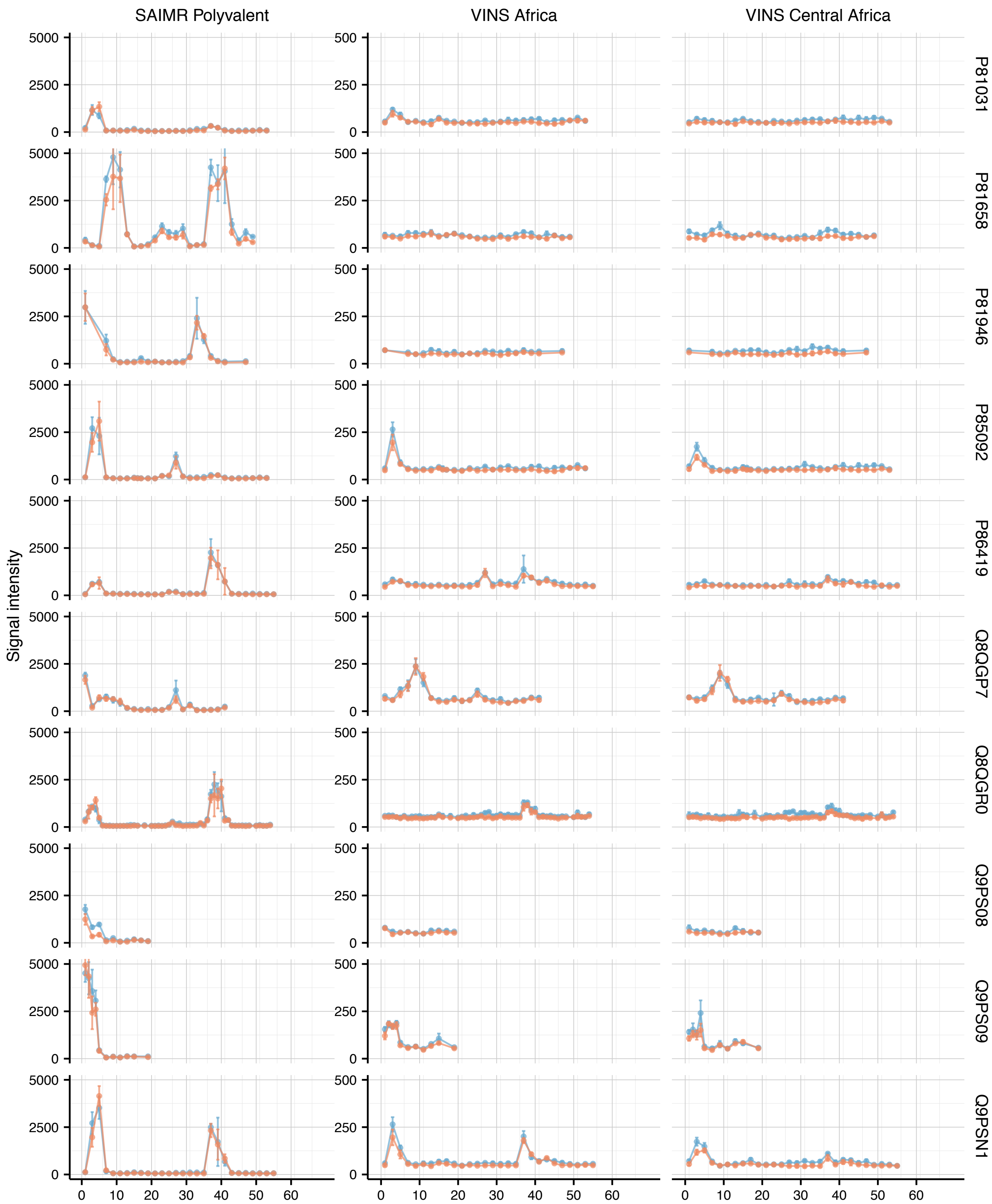

Figure S7. Binding profiles of 10 toxins. The median signal intensity of five replicates of each peptide is plotted as dots based on the position of the N-terminal residue of the 12-mer peptide in the protein sequences (pro-peptides removed). The dots are connected with straight lines to visualize the relation between overlapping peptides. The error bars represent the standard deviation of the five replicates. Positions containing gaps in the alignments were ignored. Blue dots refer to the 1:50 dilution experiment, while red dots refer to the 1:100 dilution experiments. Notice that each point contains information on the following 11 amino acids of the toxin.

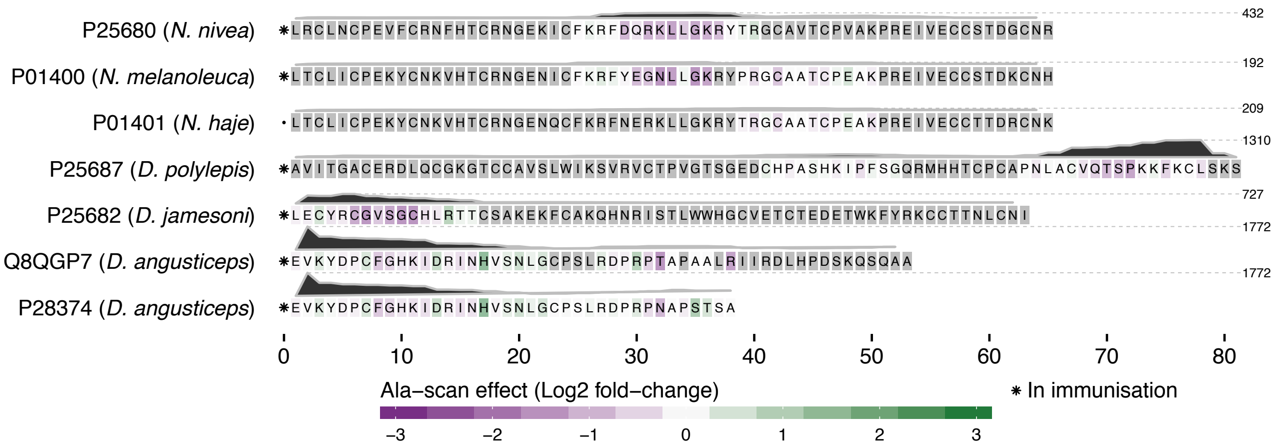

Figure S8. Linear B-cell epitope analysis of four non-conventional three-finger toxins(P01400, P01401, P25682, P25687), two Natriuretic peptides (P28374 and Q8QGP7) and one prokineticin (P25687) recognized by the SAIMR antivenom. See Fig. 4 and text for details.

Antivenom	VINS African		VINS C. Africa		SAIMR polyvalent	
Dilution	1:50	1:100	1:50	1:100	1:50	1:100
Mean (μ)	58.2	48.8	58.1	49.9	96.6	67.7
St.dev. (σ)	5.4	3.3	4.9	3.6	26.6	17.5
μ + 10σ	112.0	81.4	107.5	86.4	362.7	242.9

Table S1. Descriptive statistics on the lower 70-percentile of median signals used as an estimate of the background signal level.
